# Suicidal ideation, attempt, and its associated factors among HIV/AIDS patients in Africa: a systematic review and meta-analysis study

**DOI:** 10.1186/s13033-021-00437-3

**Published:** 2021-01-23

**Authors:** Mogesie Necho, Mekonnen Tsehay, Yosef Zenebe

**Affiliations:** Department of Psychiatry, College of Medicine and Health Sciences, Wollo University, Dessie, Ethiopia

**Keywords:** Meta-analysis, Suicide, Africa

## Abstract

**Background:**

Suicidal ideation and suicidal attempt are warning signs for and determine the prognosis of completed suicide. These suicidal behaviors are much more pronounced in people living with HIV/AIDS. Despite this, there is a scarcity of aggregate evidence in Africa. This study was therefore aimed to fill this gap.

**Methods:**

we extensively searched Psych-info, PubMed, Scopus, and EMBASE to obtain eligible studies. Further screening for a reference list of articles was also done. Meta XL package was used to extract data and the Stata-11 was also employed. Cochran’s Q- and the Higgs I^2^ test were engaged to check heterogeneity. Sensitivity and subgroup analysis were implemented. Egger’s test and funnel plots were used in detecting publication bias.

**Results:**

The pooled prevalence of suicidal ideation was 21.7% (95% CI 16.80, 26.63). The pooled prevalence of suicidal ideation in Ethiopia, Nigeria, Uganda, and South Africa was 22.7%, 25.3%, 9.8%, and 18.05% respectively. The pooled prevalence of suicidal ideation was larger; 27.7% in studies that used Composite International Diagnostic Interview (CIDI) than Mini-international Neuropsychiatric Interview (MINI); 16.96%. Moreover, the prevalence of suicidal ideation in studies with a sample size of < 400 was 23.42% whereas it was 18.3% in studies with a sample size ≥ of 400 participants. The pooled prevalence of suicidal attempts in this study was 11.06% (95% CI 6.21, 15.92). A suicidal attempt was higher in Ethiopia (16.97%) and Nigeria (16.20%) than Uganda (3.51%). This pooled prevalence of suicidal attempt was higher among studies that used a smaller sample (< 400 participants) (15.5%) than studies that used a larger sample size (≥ 400 participants) (8.4%). The pooled prevalence of suicidal attempt was 3.75%, and 16.97% in studies that used MINI and CIDI respectively. Our narrative synthesis revealed that advanced stages of AIDS, co-morbid depression, perceived HIV stigma, and poor social support was among the factors strongly associated with suicidal ideation and attempt.

**Conclusion:**

The pooled magnitude of suicidal ideation and attempt was high and factors like advanced stages of AIDS, co-morbid depression, perceived stigma, and poor social support were related to it. Clinicians should be geared towards this mental health problem of HIV patients during management.

## Background

Suicide represents 1.8% of the global disease burden and projections implied that this would increase to 2.4% in 2020 [1]. It is the 2nd among the top cause of death in a population of 15–29-years age worldwide as per data from the World Health Organization (WHO) and responsible for 71% of violent disease in women and 50% in men [2]. Epidemiologically, 85% of the global suicides rates are known to happen in low and middle-income countries (LMICS), and in Africa per year, nearly 34,000 suicides are occurring among the general population [3]. An experience with suicidal ideation and a suicidal attempt is the strongest warning sign that indicates the increased probability of completed suicide [4, 5].

HIV/AIDS is a significant public health concern and globally, more than 34 million people are living with HIV/AIDS recently [6]. Mental health and HIV/AIDS are interrelated; one affecting and predisposing to the other; mental health problems could happen due to the direct impact of HIV infection like stigma, opportunistic infections, or medication side effect and on the other side presence of mental illness increased risk of contracting HIV/AIDS and impede with its management due to poor insight and judgment [7]. Compared to the general population, people living with HIV/AIDS have 7 to 36 times greater risk of completed suicide. Suicidal ideation in HIV/AIDS is a predictor of future suicidal attempt and completed suicide and it is associated with reduced quality of life and poor adherence to antiretroviral therapy [8].

Different studies in different countries showed that suicidality is high among HIV/AIDS patients.

A systematic review and meta-analysis study on HIV/AIDS patients reported that the prevalence of suicidal ideation and attempt were 24.38% and 13.08% respectively [9]. A study in Chicago city, USA by Martinez et al. [10] screened and assessed violence and mental health disorders in HIV-positive individuals and obtained that 21.6% as having suicidal ideation. Another study in the southeastern United States obtained that suicidal ideation was found in 10% of participants [11]. A study in Thailand also revealed that suicidal ideation in HIV positive individuals was 15.5 [12].

Similarly, studies in Africa showed that suicidal ideation ranges from 8.3% to 28.8% in South Africa [13–16], 7.8% to 13% in Uganda [8, 17, 18], and 7.8% to 42% in Nigeria [19–31]. Studies in Ethiopia also reported suicidal ideation in HIV/AIDS patients to be between 22.5% and 33.6% [28–30]. The prevalence of suicidal attempt was also 9% in Japan [32], 3.5% in Canada [33], 8.2% in Thailand [12], 1.3% in Nigeria [26] and in between 13.9% and 20.1% in Ethiopia [28–30].

In the USA and Switzerland high comorbidity of depression, substance use disorders, social isolation, stigma, chronic pain, and fatigue associated with HIV/AIDS were correlates of suicidal behavior [34]. A study in Taiwan also reported that recent psychological distress, lifetime experience of depression, serious anxiety or tension, and hallucinatory experience as correlates of suicidal ideation [35]. In the context of Nigeria, a study revealed that female gender, co-morbid medical illness, unemployment, living alone, and having a partner with illness were associated factors for suicidal ideation [24]. Advanced stages of AIDS [20, 29, 30], co-morbid depression [23, 28–30], perceived HIV stigma [26, 29, 30], poor social support [18, 25, 28] were among the factors strongly and positively associated with suicidal ideation.

Furthermore, being female, WHO stage –III AIDS, presence of Opportunistic infection, comorbid depression, poor social support [30], WHO clinical stage–III and IV, being female, Not on Highly Active Anti-Retroviral therapy (HAART) and ever substance use [29], being female, being single, presence of opportunistic infection, perceived stigma and poor social support [28] were some of the associated factors for suicidal attempt in HIV/AIDS patients.

The consequences of suicidal behaviors are not merely a loss of life but extend to the mental, behavioral and emotional trauma imposed on friends and family members and costs to resources, as people with suicidal behaviors often require help from health care and psychiatric institutes [36]. Prior suicidal behaviors like suicidal ideation and attempt are among the strongest prognosticators of completed suicide [4, 5], signifying that suicidal behavior as useful outcomes of investigations.

Despite this, and the high prevalence of HIV/AIDS in Africa and the variation of the prevalence of suicidal ideation and attempt in HIV/AIDS from country to country in the context of Africa, there is no pooled evidence for suicidal ideation and attempt in HIV/AIDS patients. This creates difficulty for policymakers and researchers in decision making for the suicidal behavior of HIV patients. Therefore the present systematic review and meta-analysis study aimed and assessed [1] the magnitude of suicidal ideation and its associated factors in HIV/AIDS patients in Africa, [2] the magnitude of suicidal attempt, and its associated factors in HIV/AIDS patients in Africa.

## Methods

### Search strategy

This meta-analysis study was undertaken following the Preferred Reporting Items for Systematic Reviews and Meta-Analyses (PRISMA) guidelines [37]. The search strategy for this review has been done in two ways. The first was an exploration of electronic databases (Scopus, PubMed, Psych-Info, and EMBASE) for the presence of evidence regarding suicidal behaviors in HIV/AIDS patients. We have used the following keywords and headings for PubMed database searching: (Epidemiology OR prevalence OR magnitude AND “suicidal ideation” OR suicidality OR “suicidal attempt” AND HIV OR AIDS OR ART AND factor OR “risk factor” OR determinant AND Africa). Besides, the search for data in EMBASE, Psych-Info, and Scopus was conducted in line with database-specific searching guidelines using keywords used in PubMed. The second strategy was a manual search for the reference lists of the incorporated studies. We had not put time restrictions in our search for articles.

### Eligibility criteria’s

An article was eligible for inclusion if it meets the coming criteria’s: (1) The primary criteria were that the study had been conducted in adult HIV/AIDS patients, (2) study design was cross-sectional, cohort and case–control study design, (3) the outcome investigated should be suicidal ideation and suicidal attempt, and (4) a study should be conducted in Africa. Previous reviews, studies which assessed non-human subjects, editorials, and publication of the article in Non-English language were bases for the exclusion of article. MN and MT independently screened the titles and abstracts of articles stored in an endnote reference manager using the prespecified eligibility criteria. In the next step, these review authors read the whole content of the articles that were not excluded in the first step and independently decided on the articles that had to be included for final meta-analysis. Any differences in ideas regarding inclusion/exclusion criteria between these two authors were solved by consensus and discussion with a final reviewer (YZ).

### Methods for data extraction and quality assessment

The two formerly mentioned authors (MN and MT) extracted the relevant data from the articles included in the final analysis independently using a standardized data extracted template. The included studies were extracted and summarized in the form of a table. Information extracted and summarized in the table includes the author’s name, publication year, study setting, study population, sample size, study design, and the assessment instrument for suicidal behavior in HIV patients. Included studies were extracted for data based on the assessment template organized as suggested by PRISMA guidelines [37].

The modified Newcastle–Ottawa Scale (NOS) [38] has been used during the evaluation of the quality of studies incorporated in the final analysis. The domains of the NOS scale in assessing assesses quality includes: representativeness of sample and sample size, comparability between participants, statistical quality and ascertainment of cases.

### Data synthesis and analysis

In this meta-analysis, we employed a random-effect model to compute the pooled prevalence of suicidal ideation, attempt, and its associated factors with their 95% CIs [39]. We used Meta-XL version 5.3 [40] and STATA11 Meta-prop package [41] to undertake this meta-analysis procedure. Heterogeneity between the included studies was assessed with *Q* and *I*^2^ statistics [42]. The *I*^2^ statistical value of zero designates the absence of heterogeneity and *I*^2^ values of 25, 50, and 75% signify little, moderate, and great heterogeneity respectively [42]. Since our study was subjected to the influence of potential heterogeneity, we identified the probable source of heterogeneity using a sensitivity analysis. Moreover, the subgroup analysis based on the study setting, sample size, year of publication and the tools used to measure suicidal ideation and attempt had been performed. An eyeball funnel plot test [43] and Egger’s regression test were used to detect the presence of publication bias. Any analyses with a P-value < 0.05 was understood as statistically significant.

## Results

### Identification of studies

**Fig. 1:**
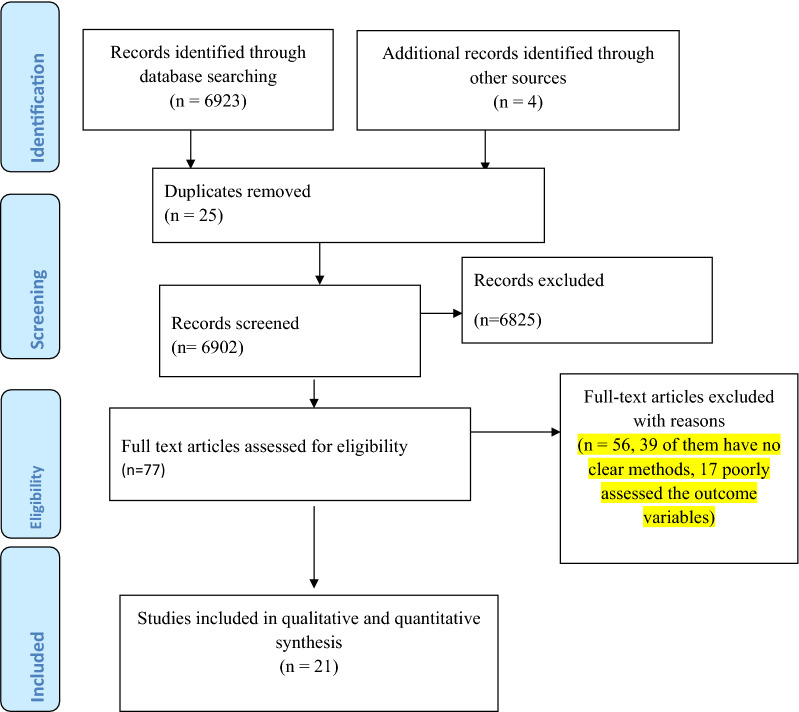
PRISMA flow chart for the review search process

Our search for literature using the previously stated search strategies resulted in a total of 6923 papers. An additional search for the reference list of documented papers also resulted in 4 articles that together give a total of 6927 articles. Among these, 25 were duplicated studies and so were excluded. About 6825 of the articles were excluded merely by looking at their titles (5256 were excluded since the title implied that the outcome variable is not suicidal ideation/attempt and the remaining 1569 were excluded since the target population is not HIV/AIDS patients). The remaining 77 articles were fully investigated for eligibility of inclusion into the meta-analysis but only 21 articles were fitted in the final meta-analysis since the rest 56 articles were also omitted because of technical and methodological constraints (Fig. 1).

### Characteristics of included studies

An overall of 21 studies that examined suicidal ideation or suicidal attempt in 7128 HIV/AIDS patients in Africa had been included in this systematic review and meta-analysis study [8, 13–22, 24–30, 44–46]. Of all included studies 11 were from Nigeria [19–31], 4 from South Africa [13–16], 3 were from Uganda [8, 17, 18], and the remaining three from Ethiopia [28–30]. The design of the studies was one cohort [13], two case–control [21, 24], and 18 cross-sectional [8, 14–20, 22, 23, 25–31]. Among all of the included studies, the assessment instruments for suicidal behaviors were CIDI in five studies [18, 23, 28–30], MINI in nine studies [8, 15–17, 21, 25–27, 31], BDI in four of the studies [13, 14, 20, 22], one PHQ-9 [19] and not specified in one study [24]. Besides, among the included studies, seven used a sample size of more than 400 [8, 18, 23, 27, 29, 30] and the remaining 14 [13, 14, 16, 17, 19–22, 24–26, 28] used a sample less than 400. Moreover, considering the year of publication of the study, nine were published in the past 5 years (after 2015) [15, 18, 23, 26–30, 44] whereas the remaining twelve were published before or in 2015 [8, 14, 16, 17, 19–22, 24, 25, 31] (Table 1).

**Table 1:** Characteristics of studies on suicidal behaviors among HIV/AIDS patients which are incorporated in the meta-analysis according to author first name, year of publication, setting of study, design, sample size, assessment instrument, study population and magnitude of suicidal ideation and attempt

Author, year	Location	Study design	Sample size	Tool used	Quality score	Outcome variables	% Suicidal ideation	Ideation cases (n)	% Suicidal attempt	Attempt cases (n)
Gebremariam et al. 2017	Ethiopia	CS	417	CIDI	9	Suicidal ideation & attempt	22.5	94	13.9	58
Bitew et al. 2016	Ethiopia	CS	393	CIDI	9	Suicidal ideation & attempt	33.6	132	20.1	79
Wondie et al. 2019	Ethiopia	CS	413	CIDI	9	Suicidal ideation & attempt	27.1	112	16.9	70
Shitu et al. 2014	Nigeria	CS	170	PHQ-9	8	Suicidal ideation	16.5	28	NA	NA
Chikezie Eze et al. 2012	Nigeria	Case control	150	Not specified	8	Suicidal ideation & attempt	34.7	52	9.3	14
Onyebueke et al. 2015	Nigeria	Case control	180	MINI	8	Suicidal ideation	7.8	14	NA	NA
Ogundipe et al. 2015	Nigeria	CS	295	Suicidal item of BDI	9	Suicidal ideation	13.6	40	NA	NA
UE chikezie et al. 2013	Nigeria	CS	150	Suicidal item of BDI	8	Suicidal ideation	42	63	NA	NA
Adeyemo et al. 2019	Nigeria	CS	201	MINI	8	Suicidal ideation	33.3	67	NA	NA
Bolakale et al. 2016	Nigeria	CS	250	MINI	8	Suicidal ideation	30	75	NA	NA
Seb-akhahomen et al. 2018	Nigeria	CS	410	MINI	9	Suicidal ideation	33.6	138	NA	NA
Bankole	Nigeria	CS	75	MINI	7	Suicidal ideation	16	12	NA	NA
Musisi et al. 2009	Nigeria	CS	82	ICD-10	7	Suicidal attempt			17.1	14
Rukundo et al. 2016	Uganda	CS	543	CIDI	9	Suicidal ideation & attempt	8.8	48	3.1	19
Kinyanda et al. 2012	Uganda	CS	618	MINI	9	Suicidal ideation & attempt	7.8	48	3.9	24
Petrushkin et al. 2005	Uganda	CS	46	MINI	7	Suicidal ideation	13	8	NA	NA
Govender et al. 2012	South Africa	Cohort	157	Suicidal intention item of BDI	7	Suicidal ideation	24.1	38	NA	NA
Schlbusch et al. 2015	South Africa	CS	189	Suicidal intention item of BDI	8	Suicidal ideation	28.8	54	NA	NA
Casale et al. 2019	South Africa	CS	1053	MINI	10	Suicidal ideation & attempt	8.3	83	4.2	44
Olley et al. 2005	South Africa	CS	149	MINI	7	Suicidal ideation	11	16	NA	NA

AIDS: Acquired Immune Deficiency Syndrome; BDI: Beck Depression Inventory; CIDI: Composite International Diagnostic Interview; CS: Cross-sectional; HIV: Human Immune Virus; ICD-10: International Classification for Disease-10; MINI: Mini-International Neuro-psychiatric Interview; PHQ-9: Patient Health Questionnaire-9; PTSD: Post Traumatic Stress Disorder; NA: Not available

### Quality of included studies

In general, the Newcastle–Ottawa quality assessment scale is used as a gold standard in the current study. The possible ranges of scores in this scale are from 0 to 10 points. The summary of quality assessment result of 21 included studies included in the present meta-analysis differs from 7 to 10. A score on this scale of 8 and above was considered as having good quality, a score of 3 to 7 moderate qualities, and less than this as poor quality. Of the 21 included studies, 16 were having good quality. Five of the included studies were also having a moderate quality but none of the studies were of poor quality.

### The pooled prevalence of suicidal ideation among HIV/AIDS patients in Africa

**Fig. 2:**
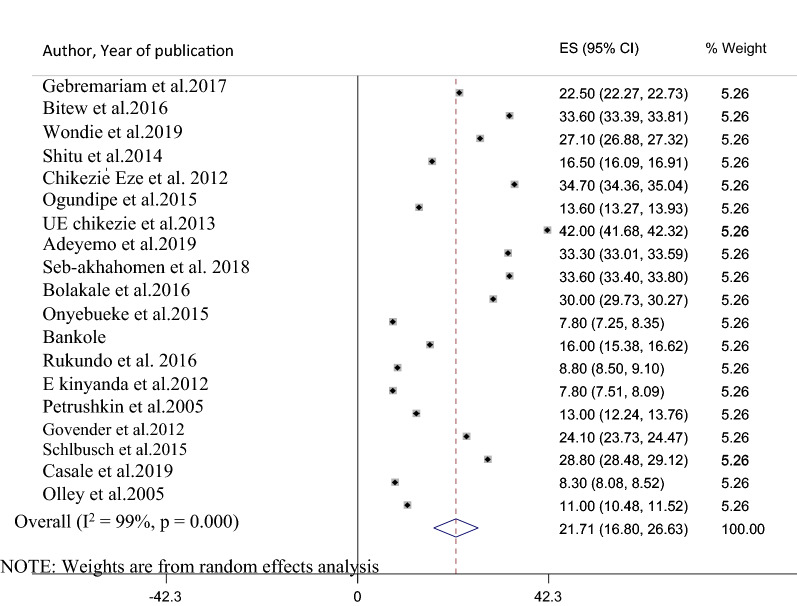
A forest plot for the prevalence of suicidal ideation in HIV/AIDS patients

Nineteen studies that assessed suicidal ideation in HIV/AIDS patients had been included in the final meta-analysis to determine the pooled prevalence of suicidal ideation. The reported magnitude of suicidal ideation among studies included in the current review and meta-analysis varies from 7.8 in Uganda and Nigeria [8, 21] to 42% in Nigeria [20]. The pooled prevalence of suicidal ideation among patients with HIV/AIDS in Africa using the random effect model was 21.7% (95% CI 16.80, 26.63). This average prevalence was under the influence of a significant heterogeneity (I^2^ = 99%, p-value ≤ 0.001) from the variance between the included studies (Fig. 2).

### Sub-group analysis of the prevalence of suicidal ideation among HIV/AIDS patients in Africa

**Fig. 3:**
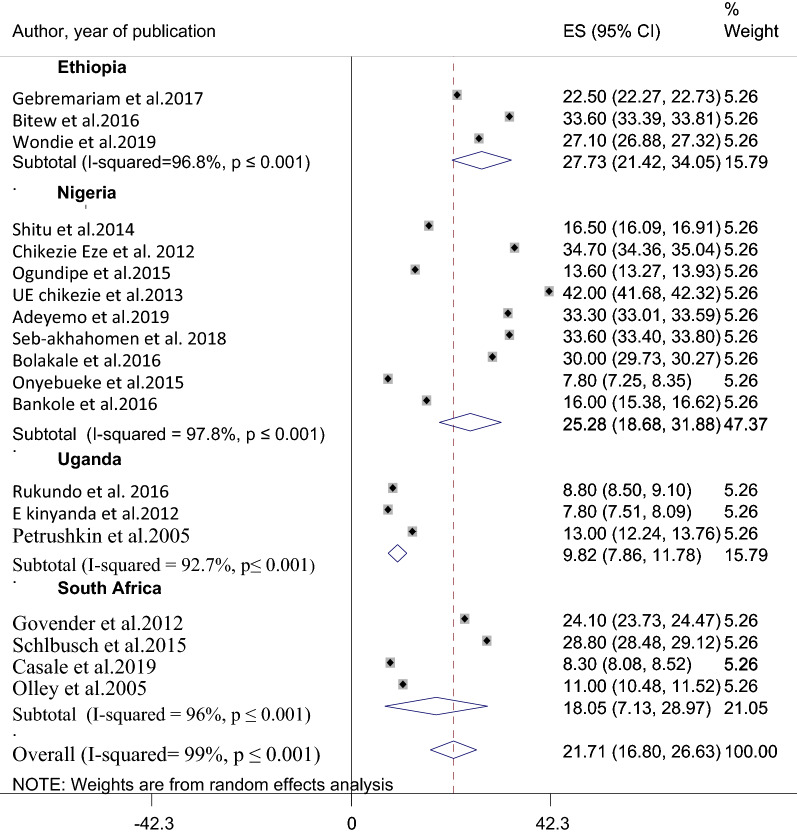
A sub-group analysis for the prevalence of suicidal ideation

Since the pooled prevalence of suicidal ideation was influenced by substantial heterogeneity, a subgroup analysis has been employed based on the study setting where the study was conducted. Therefore, among the nineteen studies included in the meta-analysis, 3 were from Ethiopia [28–30], 3 were from Uganda [8, 17, 18], 9 were from Nigeria [19–22, 24–27, 31] and four were studied in South Africa [13–16]. The pooled prevalence of suicidal ideation among HIV/AIDS patients in Ethiopia was 22.7% (95% CI 21.42, 34.05) with (I^2 ^=96.8%, p-value ≤ 0.001). The pooled prevalence of suicidal ideation in Nigeria was also obtained to be 25.3% (95% CI 18.68, 31.88) with (I^2^ = 97.8%, p ≤ 0.001). The average prevalence of suicidal ideation in Uganda and South Africa were 9.8% (95% CI: 7.86, 11.78) (I^2^ = 92.7%, p ≤ 0.001) and 18.05% (95% CI 7.13, 28.97) (I^2^ = 96%, p ≤ 0.001) respectively (Fig. 3). Besides, a subgroup analysis was done considering the assessment tool, sample size, and year of publication.

The pooled prevalence of suicidal ideation was larger; 27.7% (95% CI 21.42, 34.05) (I^2^ = 96.8%, p ≤ 0.001) in studies that used CIDI [18, 28–30] than studies that assessed suicidal ideation with MINI [8, 15–17, 21, 25–27, 31]; 16.96% (95% CI 8.97, 24.95) (I^2^ = 94.6%, p ≤ 0.001). Taking the average approximate sample size categorization of two earlier meta-analysis studies [47, 48], a sub-group analysis of suicidal ideation based on sample size was done. Moreover, the average prevalence of suicidal ideation in studies that used a sample size < 400 [13, 14, 16, 17, 19–22, 24–26, 28, 31] was 23.42% (95% CI 18.12, 28.72) (I^2^ = 98.4%, p-value ≤ 0.001) whereas it was to be 18.3% (95% CI 8.87, 27.16) (I^2^ = 96.20%, p-value ≤ 0.001) in those studies that utilized sample size ≥ 400 [8, 15, 18, 27, 29, 30] (Table 2). Last but not least suicidal ideation was relatively higher in studies published after 2015 (24.65%, 95% CI: 17.47, 31.83) than studies published in 2015 and after (19.57%, 95% CI 12.20, 26.95).

**Table 2:** A subgroup analysis of the prevalence of suicidal ideation among HIV/AIDS patients in Africa

Subgroup	Number of studies	Estimates		Heterogeneity		
		Prevalence (%)	95% CI	I^2^ (%)	Q(DF)	P-value
Country						
Ethiopia	3	27.7	21.42, 34.05	96.8	195.17(2)	< 0.001
Nigeria	9	25.3	18.68,31.88	97.8	237.2(8)	< 0.001
Uganda	3	9.80	7.86, 11.78	92.7	157.9(2)	< 0.001
South Africa	4	18.05	7.13, 28.97	96	186.23(3)	< 0.001
Study design used						
Cross-sectional	16	21.60	16.19, 27.05	98.6	521.57(15)	< 0.001
Cohort and case control	3	22.20	8.48, 35.92	98.8	632.56(2)	< 0.001
Sample size studied						
< 400	13	23.42	18.12, 28.72	98.4	498.12(12)	< 0.001
≥ 400	6	18.30	8.87, 27.16	96.2	235.05(5)	< 0.001
Assessment tool used						
MINI	10	16.96	8.97, 24.95	94.6	158.32(9)	< 0.001
CIDI	4	27.70	21.42, 34.05	96.8	195.17(2)	< 0.001
BDI and PHQ-9	5	25.00	14.86, 35.14	94.2	165.20(4)	< 0.001
Year of publication						
After 2015	8	24.65	17.47, 31.83	87	96.3(7)	< 0.001
Before and in 2015	11	19.57	12.20, 26.95	92	128.5(10)	< 0.001

BDI: Beck Depression Inventory; CI: confidence interval; CIDI: Composite International Diagnostic Interview; DF: degree of freedom; PHQ-9: Patient Health Questionnaire-9

### The pooled prevalence of suicidal attempt among HIV/AIDS patients in Africa

**Fig. 4:**
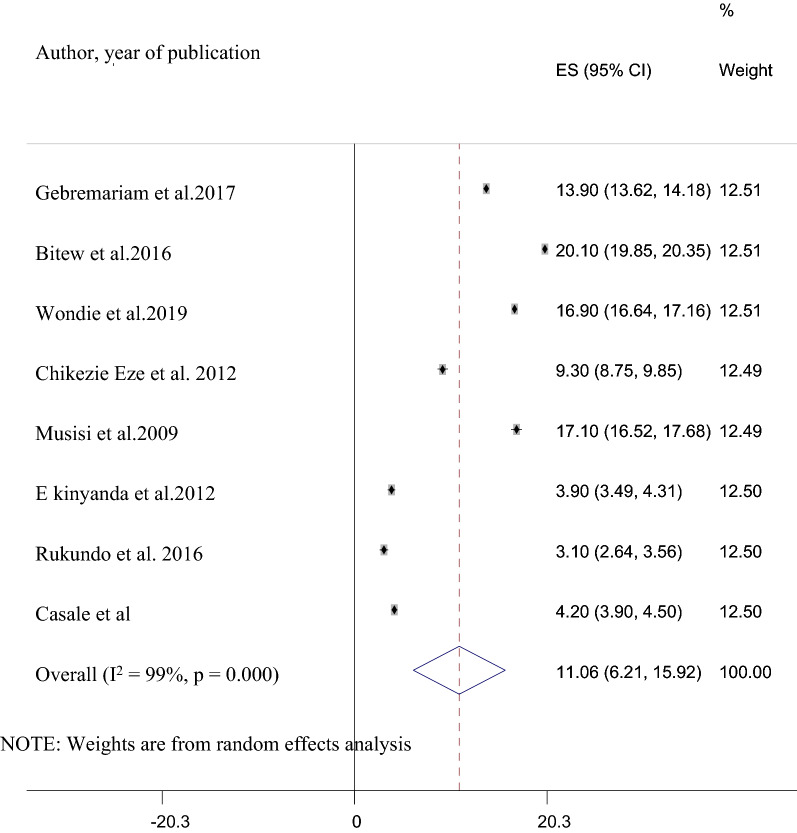
A forest plot for the prevalence of suicidal attempt in HIV/AIDS patients

Among the 21 studies included in the final analysis, data regarding suicidal attempt was reported in eight studies [8, 15, 18, 24, 28–30]. The prevalence of suicidal attempts reported in these included studies ranges from 3.1% in Nigeria [46] to 20.1% in Ethiopia [28]. The pooled prevalence of suicidal attempts in these studies was 11.06% (95% CI 6.21, 15.92). This pooled prevalence was also having a substantial heterogeneity (I^2^ = 99%, P ≤ 0.001) (Fig. 4).

### Subgroup analysis of the prevalence of suicidal attempt

Since the pooled prevalence of suicidal attempt was also influenced by substantial heterogeneity, a subgroup analysis based on study country, sample size studied, measurement instrument used for the suicidal attempt, and year of study was implemented. The average prevalence of suicidal attempt in Uganda was 3.51% (95% CI 2.72, 4.29) and was very much lower than the average prevalence of suicidal attempt in Nigeria; 13.20 (95% CI 5.56, 20.84) and Ethiopia [28–30]; 16.97% (95% CI 13.48, 20.46). The pooled prevalence of suicidal attempt among studies that used relatively smaller sample (< 400 participants) [24, 28] was larger; 15.5% (95% CI 9.06, 21.95) than the pooled prevalence of studies that used a larger sample size (≥ 400 participants) [8, 15, 18, 29, 30] which was 8.4% (95% CI 2.52, 14.29). The pooled prevalence of suicidal attempt among studies assessed with MINI [8, 15] was 3.75% (95% CI 3.13, 4.37) and was smaller than the pooled prevalence of suicidal attempt in studies assessed by CIDI [18, 28–30]; 16.97% (95% CI 13.48, 20.46). Moreover, the pooled prevalence of suicidal attempt was higher in studies done after 2015 [15, 28–30, 46]; 13.5% (95% CI 7.94, 19.07) than the pooled prevalence of studies done before 2015 [8, 24]; 8.6% (95% CI 3.30, 13.92) (Table 3).

**Table 3:** A subgroup analysis of the prevalence of suicidal attempt among HIV/AIDS patients in Africa with its 95% confidence interval

Subgroup	Number of studies	Estimates		Heterogeneity		
		Prevalence (%)	95% CI	I^2^ (%)	Q(DF)	P-value
Country						
Ethiopia	3	16.97	13.48, 20.46	94.2	167.4(2)	< 0.001
Nigeria	2	13.20	5.56, 20.84	92,4	123.4(1)	< 0.001
Uganda	2	3.51	2.72, 4.29	96.6	456(1)	< 0.001
Assessment tool used						
MINI	2	3.75	3.13, 4.37	87.1	85.27(2)	< 0.001
CIDI	4	16.97	13.48, 20.46	99.8	1079.2(8)	< 0.001
Others	2	13.2	5.56, 20.84	96.7	379.6(2)	< 0.001
Sample size studied						
< 400	3	15.5	9.06, 21.95	98.4	768.12(12)	< 0.001
≥ 400	5	8.4	2.52, 14.29	98.2	635.05(4)	< 0.001
Year of publication						
Before 2015	3	8.6	3.30, 13.92	98.6	785.32(3)	< 0.001
After 2015	5	13.5	7.94, 19.07	98.8	1065.17(3)	< 0.001

### Sensitivity analysis

We performed a sensitivity analysis to detect the source of heterogeneity that affects the pooled prevalence of suicidal ideation in HIV/AIDS patients. The result from the sensitivity analysis revealed that the pooled estimated prevalence of suicidal ideation obtained when every single study was left out from analysis was within the 95% confidence interval of the pooled prevalence of suicidal ideation when all studies were fitted together. Moreover, the sensitivity analysis result showed that the pooled prevalence of suicidal ideation ranges between 20.58 (95% CI 15.76, 25.71) and 23.85% (95% CI 23.78, 23.92) when each study was left out from the analysis (Table 4). Also, we did a sensitivity analysis for the prevalence of suicidal attempt but none of the individual studies out-weighted the average prevalence of suicidal attempt (Table 5).

**Table 4:** A sensitivity analysis of the prevalence of suicidal ideation among HIV/AIDS patients in Africa when each indicated studies are removed at a time with its 95% confidence interval

No.	Study excluded	Prevalence of suicidal ideation (%)	95% Confidence interval	Remark
1	Gebremariam et al. 2017	21.67	16.37,26.96	
2	Bitew et al. 2016	21.05	15.96, 26.14	
3	Wondie et al. 2019	23.85	23.78, 23.92	
4	Shitu et al. 2014	22	16.91,27.09	
5	Chikezie Eze et al. 2012	20.99	15.93, 26.05	
6	Ogundipe et al. 2015	22.16	17.11, 27.22	
7	Chikezie et al. 2013	20.58	15.76, 25.71	
8	Adeyemo et al. 2019	21.07	15.97, 26.16	
9	Seb-akhahomen et al. 2018	21.05	15.96, 26.14	
10	Bolakale	21.25	16.07, 26.43	
11	Onyebueke et al. 2015	22.48	17.25,28.23	
12	Bankole	22.03	16.96, 27.09	
13	Rukundo et al. 2016	22.43	17.54, 27.32	
14	E kinyanda et al. 2012	22.48	17.65, 27.32	
15	Petrushkin et al. 2005	22.19	17.14, 27.25	
16	Govender et al. 2012	21.58	16.43, 26.73	
17	Schlbusch et al. 2015	21.32	16.16, 26.48	
18	Casale et al. 2019	22.46	17.79, 27.12	
19	Olley et al. 2005	22.31	17.26, 27.34

**Table 5:** A sensitivity analysis of the prevalence of suicidal attempt among HIV/AIDS patients in Africa when each indicated studies are removed at a time with its 95% confidence interval

No.	Study excluded	Prevalence of suicidal attempt (%)	95% Confidence interval	Remark
1	Gebremariam et al.2017	10.66	4.83, 16.48	
2	Bitew et al.2016	9.77	5.04, 15.44	
3	Wondie et al.2019	10.23	4.61, 15.84	
4	Chikezie Eze et al. 2012	11.31	6.00, 16.14	
5	Musisi et al.2009	10.20	4.91, 15.49	
6	Rukundo et al. 2016	12.20	7.23, 17.17	
7	Kinyanda et al. 2012	12.1	7.10, 17.07	
8	Casale et al. 2019	12.04	7.32, 16.76	

### Publication bias

**Fig. 5:**
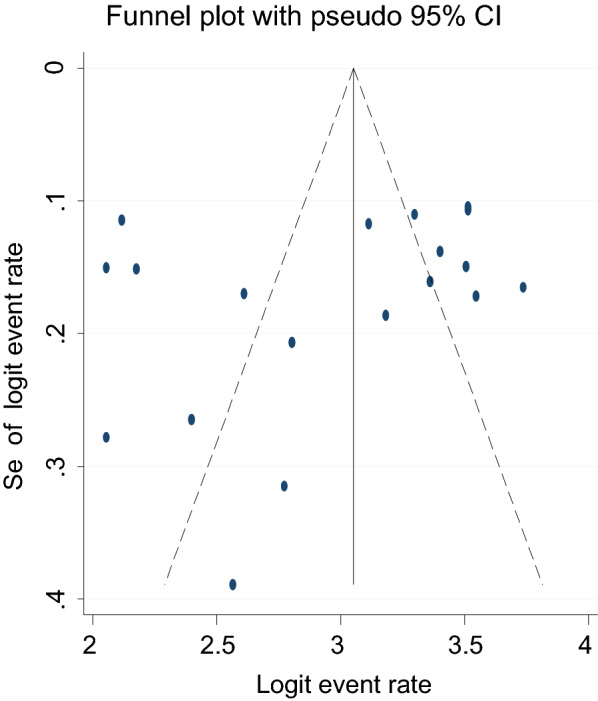
A funnel plot for the prevalence of suicidal ideation in HIV/AIDS patients

**Fig. 6:**
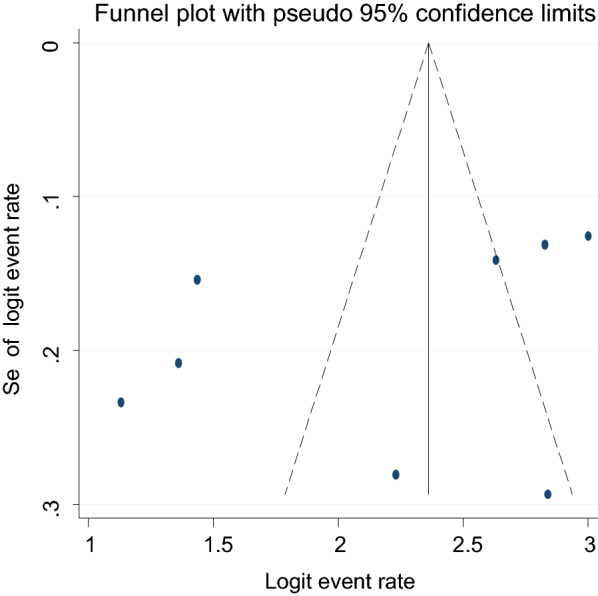
A funnel plot for the prevalence of suicidal attempt in HIV/AIDS patients

The presence/absence of publication bias for the prevalence of suicidal ideation and attempt was checked with two methods. The first was an egger’s publication bias plot. The result from this showed that the publication bias is near the origin and its p-value is non-significant; (P-value = 0.23) for suicidal ideation and (P-value = 0.22) for suicidal attempt implying that no significant publication bias for the prevalence suicidal ideation and attempt in Africa. Moreover, a visual inspection from a funnel plot for a Logit event rate of prevalence of suicidal ideation and attempt in HIV/AIDS patients against its standard error suggests supportive evidence for the absence of publication bias (Figs. 5 and 6).

### Associated factors for suicidal ideation and suicidal attempt in HIV/AIDS patients in Africa

Of all included studies, ten studies reported the factors associated with suicidal ideation [13, 18, 20, 23–26, 28–30]. A study in Nigeria revealed that female gender, co-morbid medical illness, unemployment, living alone, and having a partner with illness were associated factors for suicidal ideation [24]. Advanced stages of AIDS [20, 29, 30], co-morbid depression [23, 28–30], perceived HIV stigma [26, 29, 30], poor social support [18, 25, 28] were among the factors strongly and positively associated with suicidal ideation. Furthermore, being not on HAART, family history of suicidal attempt [29], being female and family death [30], being female, being divorced, being single, and CD4 count ≤ 500 [28], opportunistic infection, state anger, trait anger and anxiety [18], HIV non-disclosure, polygamous family, physical and emotional abuse, primary school and a decline in academic performance [25] were also among the factors associated with suicidal ideation in patients with HIV/AIDS in Africa (Table 6). Regarding suicidal attempt; female gender [28–30], WHO clinical stage-III and IV [29, 30], presence of opportunistic infection [28, 30], comorbid depression [8, 30], poor social support [28, 30] were the commonly cited associated factors. Besides, being not on HAART and ever substance use [29], being single, perceived stigma [28], perception of poor health, physical pain, reducing pain due to illness, and recent HIV diagnosis [18], high negative coping style, history of psychiatric illness, psychosocial impairment [8] were also among the factors associated with suicidal attempt (Table 7).

**Table 6:** Characteristics of associated factors for suicidal ideation among HIV/AIDS patients in Africa

Associated factors	Odds ratio (AOR)	95% CI	Strength of association	Author, year of publication
WHO-clinical stage 4	6.5	2.35, 18.2	Strong and positive	Gebremariam et al. 2017
WHO-clinical stage 3	4.12	2.07, 8.16	Strong and positive	Gebremariam et al. 2017
Not on HAART	2.39	1.07, 5.70	Moderate and positive	Gebremariam et al. 2017
Family history of suicidal attempt	2.3	1.01,5.03	Moderate and positive	Gebremariam et al. 2017
Comorbid depression	2.45	1.45, 4.12	Moderate and positive	Gebremariam et al. 2017
Perceived stigma	1.76	1.02, 3.03	Moderate and positive	Gebremariam et al. 2017
Being female	3.1	1.60, 6.00	Strong and positive	Wondie et al. 2018
Family death	2.1	1.15, 3.85	Moderate and positive	Wondie et al. 2018
WHO clinical stage-III	3.1	1.30, 7.35	Strong and positive	Wondie et al. 2018
WHO clinical stage-IV	4.8	1.8, 18.8	Strong and positive	Wondie et al. 2018
Comorbid depression	7.14	3.90, 12.9	Strong and positive	Wondie et al. 2018
Perceived HIV stigma	4.2	2.27, 8.20	Strong and positive	Wondie et al. 2018
Being female	2.6	1.30, 5.20	Moderate and positive	Bitew et al. 2016
Poor social support	2.5	1.30,4.90	Moderate and positive	Bitew et al. 2016
Being single	13.5	4.70, 39.1	Strong and positive	Bitew et al. 2016
Being divorced	2.7	1.3, 4.7	Moderate and positive	Bitew et al. 2016
CD4 count ≤ 500	2.5	1.3, 4.9	Moderate and positive	Bitew et al. 2016
Co-morbid depression	17	8.8, 33.3	Strong and positive	Bitew et al. 2016
Opportunistic infection	5.2	2.50, 10.9	Strong and positive	Bitew et al. 2016
State anger	1.1	1.03, 1.09	Weak and positive	Rukundo et al. 2016
Trait anger	1.1	1.04, 1.16	Weak and positive	Rukundo et al. 2016
Depression	1.13	1.07, 1.20	Weak and positive	Rukundo et al. 2016
Anxiety	1.1	1.03, 1.09	Weak and positive	Rukundo et al. 2016
Poor social support	0.19	0.07, 0.47	Weak and negative	Rukundo et al. 2016
Hopelessness	1.12	1.02, 1.23	Weak and positive	Rukundo et al. 2016
Comorbid infection	X^2^ = 24.08, p = 0.004		Strong and positive	UE chikezie et al. 2013
Age group 18 to 27	X^2^ = 18.88, p = 0.001		Strong and positive	UE chikezie et al. 2013
Being female	X^2^ = 9.88, p = 0.001		Strong and positive	UE chikezie et al. 2013
Stage 3 and 4 AIDS	X^2^ = 33.85, p = 0.002		Strong and positive	UE chikezie et al. 2013
Recent diagnosis	X^2^ = 30.17, p = 0.015		Strong and positive	UE chikezie et al. 2013
HIV non-disclosure	P-value = 0.0021		Strong and positive	Adeyemo et al. 2019
Physical and emotional abuse	P-value = 0.0009		Strong and positive	Adeyemo et al. 2019
Gender	P-value = 0.025		Strong, positive	Adeyemo et al. 2019
Primary school	P-value = 0.017		Strong and positive	Adeyemo et al. 2019
Polygamous family	P-value = 0.040		Strong and positive	Adeyemo et al. 2019
Poor social support	P-value = 0.031		Strong, positive	Adeyemo et al. 2019
Decline in academic performance	P-value = 0.005		Strong and positive	Adeyemo et al. 2019
Loss of family member	P-value = 0.007		Strong and positive	Adeyemo et al. 2019
Discrimination	P-value = 0.040		Strong and positive	Adeyemo et al. 2019
Being separated	3.05	1.67, 5.57	Strong and positive	Egbe et al. 2017
Never married	1.9	1.22, 3.09	Moderate and positive	Egbe et al. 2017
Divorced	2.66	1.07, 6.63	Moderate and positive	Egbe et al. 2017
Major depressive disorder	1.8	129, 2.62	Moderate and positive	Egbe et al. 2017
Being Christians	2.4	1.36, 4.26	Moderate and positive	Egbe et al. 2017
Over all physical health	0.49	0.29,0.84	moderate and negative	Egbe et al. 2017
Perceive HIV stigma	p-value < 0.001		Strong and positive	Bolakale et al. 2016
Depression	p-value < 0.001		Strong and positive	Bolakale et al. 2016
Non-educated	19.7	2.89, 133.82	Strong and positive	Govender 2012
Grade 8 attained	5.5	1.18, 26.02	Strong and positive	Govender 2012
Grade 10 attained	5.4	1.27, 23.04	Strong and positive	Govender 2012
Grade 12 attained	4.7	1.12, 20.19	Strong and positive	Govender 2012
Traditional African believes	22.6	4.25, 120.57	Strong and positive	Govender 2012

**Table 7:** Characteristics of associated factors for suicidal attempt among HIV/AIDS patients in Africa

Associated factors	Odds ratio (AOR)	95% confidence interval	Strength of association	Author, year of publication
Being female	4.1	1.8, 9.8	Strong and positive	Wondie et al. 2018
WHO clinical stage-III	3.1	1.2,7.8	Strong and positive	Wondie et al. 2018
Presence of opportunistic infections	3.1	1.6, 6.00	Strong and positive	Wondie et al. 2018
Comorbid depression	5.6	2.8, 11.1	Strong and positive	Wondie et al. 2018
Poor social support	3.4	1.20, 9.40	Strong and positive	Wondie et al. 2018
WHO-clinical stage 4	10.98	3.56, 33.79	Strong and positive	Gebremariam et al. 2017
WHO-clinical stage 3	4.46	1.93, 10.29	Strong and positive	Gebremariam et al. 2017
Being female	4.48	1.85, 10.29	Strong and positive	Gebremariam et al. 2017
Not on HAART	3.44	1.33, 8.89	Strong and positive	Gebremariam et al. 2017
Ever-substance use	3.39	1.32,8.73	Strong and positive	Gebremariam et al. 2017
Comorbid depression	2.04	1.07, 3.87	Strong and positive	Gebremariam et al. 2017
Being female	2.8	1.30, 6.20	Strong and positive	Bitew et al. 2016
Being single	8.4	3.1, 22.8	Strong and positive	Bitew et al. 2016
Presence of OI	2.3	1.20,4.80	Strong and positive	Bitew et al. 2016
Perceived stigma	2.9	1.40, 5.90	Strong and positive	Bitew et al. 2016
Poor social support	3	1.60,5.9	Strong and positive	Bitew et al. 2016
Perception of poor physical health	2.2	1.23, 3.99	Strong and positive	Rukundo et al. 2016
Physical pain	1.8	1.01, 3.30	Strong and positive	Rukundo et al. 2016
Reducing work due to illness	2.2	1.23, 3.99	Strong and positive	Rukundo et al. 2016
Recent HIV diagnosis	1.02	1.01, 1.03	weak and positive	Rukundo et al. 2016
High negative coping style score	2.5	1.93, 6.93	Strong and positive	Kinyanda et al. 2012
Past history of psychiatric illness	4.5	1.33, 15.10	Strong and positive	Kinyanda et al. 2012
Psychosocial impairment	2	1.07, 3.76	Strong and positive	Kinyanda et al. 2012
Comorbid depression	30.3	14.40, 63.80	Strong and positive	Kinyanda et al. 2012

## Discussion

To the investigator’s knowledge, this review and meta-analysis on suicidal ideation, attempt, and their associated factors in individuals living with HIV/AIDS in Africa were the first of its type. Consequently, the evidence obtained from this meta-analysis on the pooled prevalence and related factors for suicidal ideation and attempt will be significant evidence to diverse stakeholders.

This review and meta-analysis study incorporated twenty-one studies that were conducted between 2005 and 2019. Among these studies, nineteen assessed suicidal ideation as a primary outcome variable, and eight assessed suicidal attempts. The pooled prevalence of suicidal ideation among the included studies was 21.7% (95% CI 16.80, 26.63). This was in line with the result of a systematic review and meta-analysis study on HIV/AIDS patients which reported that the worldwide prevalence of suicidal ideation as 24.38% [9]. Another meta-analysis study on suicidal thoughts of college students by Mortier 2018 also obtained a consistent result; 22.3% [49]. However, this was higher than studies in the United States; 10% [11], 14.0% in Canada [33], and Thailand that also revealed that suicidal ideation in HIV positive individuals was 15.5 [12]. On the other hand, the pooled prevalence in the present study was lower than the result of studies in China; 31.6 and 64% in China [50, 51]. Differences in socio-economic and cultural differences could bring the variation. Moreover, the current study is a meta-analysis study that might provide a more precise result than the Chinese studies which are single studies. In addition to this, the result of the current meta-analysis is also lower when compared with the average prevalence of suicidal ideation in homeless people in Ethiopia which was 41.6% [52]. Homeless people are at extremely vulnerable groups and suffer from severe psychosocial and economic problems that might heighten their risk of suicidal ideation [53].

The prevalence of suicidal ideation showed a variation based on the country of study, measurement tool, study design, the sample size of the study, and year of publication. Our subgroup analysis showed that the pooled prevalence of suicidal ideation among HIV/AIDS patients was 22.7% in Ethiopia, 25.3% in Nigeria both of which were higher than the average prevalence of suicidal ideation in Uganda; 9.8%, and South Africa; 18.05%. Differences in culture regarding suicide and its stigma among the above countries will be responsible for this variation. Moreover, the difference in the number of included studies in each country could bring variation.

Considering measurement tools for suicidal ideation, a larger prevalence of suicidal ideation was obtained in studies that used CIDI (27.7%) than studies that assessed with MINI (16.97%). The difference in sensitivity and specificity of these measurement tools could bring the difference.

Besides, suicidal ideation was higher (23.42%) in studies with a relatively smaller sample size (< 400 participants) than the prevalence of studies with a small sample size (> 400 participants) (18.3%). The probability of minimization of a standard error with the increment of sample size would be responsible for the variation [54, 55].

Last but not least suicidal ideation was relatively higher in studies published after 2015 (24.65%) than studies published in 2015 and after (19.57%). This could be due to the rising awareness of the community towards suicide and their stigmas consequently increment its reporting.

The pooled prevalence of suicidal attempts in the current study was 11.06% (95% CI 6.21, 15.92). A systematic review and meta-analysis study by Tsegaye [9] on HIV/AIDS patients reported that the worldwide prevalence of suicidal attempt as 13.08% which is consistent with the present finding. In addition, this pooled prevalence of suicidal attempts was in line with the prevalence of suicidal attempts in Japan which was 9% [32], and 8.2% in Thailand [12]. However, this result was higher than the result in studies in Canada; 3.5% [33], and 1.3% in Nigeria [26]. On the contrary, the result of this study was very much lower than the pooled prevalence of suicidal attempts in homeless people in Ethiopia [52]. The current finding is also lower than a study in china; 22.6% [50] and 23% in France [56]. The variation in disclosure status of suicidal attempt attributed to socio-economic and environmental factors between the above-mentioned studies and African studies included in this analysis might bring the difference. Moreover, the presented study is a meta-analysis study and the comparator studies mentioned above are single studies that might also cause variation in the magnitude of suicidal attempt. A suicidal attempt had also shown differences based on country of study, measurement tool, sample size, and year of publication. Reasons discussed for suicidal ideation could also be applicable suicidal attempts.

Advanced stages of AIDS [20, 29, 30] were associated factors for suicidal ideation and attempt. This was supported by an earlier study [28]. The reason for this might be the deterioration of clinical conditions and rising of AIDS-defining opportunistic medical illness which further puts additional burdens and decreases the quality of life of the patients [28–30]. Further worsening of symptoms might also install hopelessness in the patient and increases suicidality.

Co-morbid depression [23, 28–30] was also positively and strongly associated with suicidality in HIV/AIDS patients in Africa. This was also strengthened by earlier studies in Japan [28] and China [50]. The reason for this is that depression will deplete the level of serotonin in our brain and studies showed that a decrease in serotonin would have an impact on suicidal behavior [36, 57]. Furthermore, the direct social impacts of depression such as social detachment, hopelessness, and worthless could be responsible.

Besides, Perceived HIV stigma [26, 29, 30] was also significantly and positively associated with suicidality in HIV/AIDS patients. Studies in China [50], and America [58] supported this conclusion. The justification for this might be may be due to the fact the feeling of being stigmatized subsidize to frequent psychological distress and finally to suicidal behaviors.

Having a good social support system is protective of any type of psychiatric illness. In this study, poor social support [18, 25, 28] were obtained to be a strong predictor for suicidality. This was strengthened by Studies in France [56]. HIV/AIDS patients who have poor social support may face difficulty in adjusting to this very support demanding chronic illness and its psychological burden by themselves and feel detached to the extent of increasing their suicidal risk [59].

Furthermore, our narrative analysis showed that other factors such as being not on HAART, family history of suicidal attempt [29], being female and family death [30], being female, being divorced, being single, and CD4 count ≤ 500 [28], opportunistic infection, state anger, trait anger and anxiety [18], HIV non-disclosure, polygamous family, physical and emotional abuse, primary school and a decline in academic performance [25] ever substance use [29], perception of poor health, physical pain, reducing pain due to illness, and recent HIV diagnosis [18], High negative coping style score, history of psychiatric illness, psychosocial impairment and comorbid depression [8] were among the factors associated with suicidality in HIV patients in Africa.

## Implications of the findings to future decision making

The results of this meta-analysis on suicidal ideation and attempt on HIV/AIDS patients have potential implications for various stakeholders for important decision making. The high pooled magnitude of suicidal ideation and attempt in the target population reported in this study relative to the general population will be a motive for further research to explore more of why this happens and what factors are correlated with this. Second, as a mental illness in general and suicidal behaviors, in particular, are not given much attention, the present finding would be important evidence for clinical practitioners working on anti-retroviral treatment centers to appropriately intervene in this population. Last but not least, program planners and policymakers would design appropriate programs and plans for an integrated approach in the management of individuals living with HIV/AIDS.

## Strengths and limitations of the study

This review and meta-analysis have their strengths and limitations. Its strength begins with the use of a prespecified search strategy that minimizes the reviewer’s bias. The second strength was that the data extraction and quality assessment of the study was done by independent reviewers that also further minimize the reviewer’s bias. The implementation of subgroup analysis and sensitivity analysis to detect the source of heterogeneity was a strength. On the contrary, the limitations of the present study rise from the existence of heterogeneity that might affect the conclusion of the study findings. Another limitation is that the inclusion of a few numbers of studies in the subgroup analysis might minimize the validity of the estimate.

## Conclusion and recommendation

This meta-analysis study revealed the high prevalence of suicidal ideation and attempt among HIV/AIDS patients in Africa and it is significantly related to advanced stages of AIDS, co-morbid depression perceived HIV stigma, and poor social support. Hence, to improve the quality of life of people living with HIV, much consideration has to be given to reduce these suicidal behaviors and modify the associated factors basically by integrating mental health services into the routine anti-retroviral therapy for patients. Further studies are suggested to incorporate larger samples of participants and to conduct follow up of studies.

## Data Availability

All relevant data regarding this research work is included in the manuscript.

## Abbreviations

AIDS: Acquired Immune-Deficiency Syndrome
BDI: Beck Depression Inventory
CI: Confidence Interval
CIDI: Composite International Diagnostic Interview
DF: Degree of Freedom
HIV: Human Immune Virus
PHQ-9: Patient Health Questionnaire-9
